# Corrosion Behavior of Pressure Infiltration Diamond/Cu Composites in Neutral Salt Spray

**DOI:** 10.3390/ma13081847

**Published:** 2020-04-14

**Authors:** Zhongnan Xie, Hong Guo, Ximin Zhang, Shuhui Huang

**Affiliations:** 1 State Key Laboratory of Nonferrous Metals and Processes, GRINM Group Co., Ltd., Beijing 101400, China; zhongnanx@126.com (Z.X.); zxmbeibei@126.com (X.Z.); hithuang@126.com (S.H.); 2 GRIMAT Engineering Institute Co., Ltd., Beijing 101400, China; 3 General Research Institute for Nonferrous Metals, Beijing 100088, China

**Keywords:** metal matrix composites, Diamond/Cu composites, neutral salt spray, corrosion behavior, thermal conductivity

## Abstract

Diamond particle-reinforced copper matrix composites (Diamond/Cu) are recognized as promising electronic packaging materials due to their excellent thermophysical properties. It is necessary to investigate the reliability of Diamond/Cu composites under extreme environmental conditions. The corrosion behavior of Diamond/Cu composites was studied in a 5 wt% NaCl neutral salt spray. Surface morphology, thermal conductivity, bending strength, corrosion rate, and corrosion depth resulting from corrosion were researched in this paper. The results showed that the corrosion phenomenon mainly occurs on the copper matrix, and the diamond and interface products do not corrode. The corrosion mechanism of Diamond/Cu composites was micro-galvanic corrosion. The corrosion product formed was Cu_2_Cl(OH)_3_. The salt spray environment had a great influence on the composite surface, but the composite properties were not significantly degenerated. After a 168-h test, the bending strength was unaltered and the thermal conductivity of gold-plated composites showed a slight decrease of 1–2%. Surface gold plating can effectively improve the surface state and thermal conductivity of Diamond/Cu composites in a salt spray environment.

## 1. Introduction

Due to their high thermal conductivity and adjustable thermal expansion coefficient, Diamond/Cu composites have great potential for heat sinks and base plates in high performance electronic packaging [1,2]. Compared with current commercial thermal management materials, such as W-Cu [3], Mo-Cu [4], SiC/Al [5], etc., Diamond/Cu composites have outstanding performance advantages in thermal conductivity (>600 W/mK) [6,7]. Electronic packaging materials should possess not only high thermal conductivity, but also reliability and stability of performance. It is necessary to investigate the reliability and performance evolution of Diamond/Cu composites under extreme environmental conditions before further application.

Metal matrix composites (MMCs) are usually composed of metal matrices and reinforcements with different physical and chemical properties. In a neutral salt spray environment, the metal matrix and reinforcement exhibit different electrochemical corrosion potentials and corrosion characteristics. The corrosion mechanism of MMCs is mainly in the following forms [8,9,10]. Compared with monolithic matrix alloys, the introduction of the reinforcement phase changes the homogeneity of structure and composition, making the matrix more susceptible to localized corrosion. Interfacial reaction products, impurities, defects, etc., all affect the corrosion resistance of the composites. The corrosion behavior of MMCs is also affected by the formation of interfacial products in the process of fabrication [11,12]. Due to the mismatch of the thermal expansion coefficient between the reinforcement and matrix, there will be stress and high dislocation density at the interface, which will accelerate corrosion [8].

Corrosion behavior of copper matrix composites reinforced with diamond, SiC, graphite, and graphene has been reported [13,14,15,16,17]. Due to the difference of the reinforcement phase, the corrosion behavior of composite materials is different. In the 3D-SiC-reinforced copper matrix composites, copper corrosion at the matrix interface is severe. This position is liable to form a corroded galvanic cell because of uneven chemical properties and high residual stress. Graphite/Cu composites have better corrosion resistance than copper. Corrosion occurs at grain boundaries, rather than at the interface between the graphite and copper matrix [16]. However, some scholars believe that graphite has a more noble potential than copper, and the galvanic coupling in the sample leads to the increase of the local corrosion rate. The corrosion resistance of Graphene/Cu composites is related to the arrangement of graphene. Transversely arranged graphene can greatly improve the corrosion resistance of the composite [15]. In addition to the types of reinforcements, the corrosion behavior of composites is also affected by the reinforcement content. It has been found that the corrosion rate of the composites decreases with the increase of the content of reinforcing materials [18,19].

A great deal of work has been done on the thermophysical and mechanical properties of Diamond/Cu composites. However, systematic work on the corrosion behavior and property evolution of Diamond/Cu composites has not yet been carried out. We used 60 vol% and 75 vol% Diamond/Cu composites to carry out salt spray tests, mainly because these two types of composites have excellent comprehensive properties, such as high thermal conductivity, outstanding mechanical properties, and a semiconductor-matched thermal expansion coefficient. They are the two types of Diamond/Cu composites with the greatest potential for large-scale application in the future. In view of this, the purpose of this study was to investigate the effect of corrosion on the surface condition, microstructure, and properties of Diamond/Cu composites during a neutral salt spray test. Based on analyzing the corrosion rule of Diamond/Cu composites, feasible anticorrosion improvement is put forward in order to provide guidance for the future application of this type of material.

## 2. Materials and Methods

### 2.1. Materials

Diamond/Cu composites were prepared by pressure infiltration. The diamond sizes in 60 vol% Diamond/Cu composites were 100 μm, and 75 vol% Diamond/Cu composites were 50 μm and 400 μm mixed. In order to prepare Diamond/Cu composites, Cu-Cr alloy was melted and poured into the preform. The details of the pressure infiltration can be referred to elsewhere [6,7].

Ni-Au was plated on the surface of the Diamond/Cu composites by electroplating. The coating consisted of a 5 μm Ni layer and 3 μm Au layer to ensure no copper or diamond was exposed. The mechanically polished Diamond/Cu composites were sensitized and activated in a sensitizing activation solution. Nickel plating was carried out using electroplating equipment at 82 °C and pH 4.5 for 20 min, and Au was deposited using Di-propanedinitrile gold-based solution at 52 °C and pH 5 for 15 min. The specific process is reported in reference [20].

### 2.2. Salt Spray Corrosion Test

Neutral salt spray experiments were carried out in a sealed test chamber (Scch-21, Singleton, Cleveland, OH, USA). The polished samples were cleaned with anhydrous ethanol and deionized water in turn, and then placed in the same horizontal position to ensure the same amount of corrosive medium. The sample was exposed to a neutral salt spray environment in accordance with the national standard GB/T 2423.17-93 [21]. The specific experimental conditions were: pH 6.5–7.2; salt spray was from a neutral 5 wt% NaCl solution and was provided in a continuous manner at 35 °C; the deposition rate was 1.5–1.6 mL/h; the samples’ corrosion times were 16, 24, 48, 96, 168 h, respectively. The sedimentation rate was determined using the specific method in accordance with GB/T 2423.17-93. At any position in the salt spray box, a funnel with an area of 80 cm^2^ could collect 1.5 to 1.6 mL solution per hour. From this, a deposition rate of 1.5 to 1.6 mL/h was determined. The sample collection surface was placed horizontally in the salt spray box. Each sample was at the same distance from the salt spray generator.

### 2.3. Corrosion Weight Loss Test

The mass loss of Diamond/Cu composites sample was tested using an electronic balance (ME204T/02, 0.1 mg, METTLER TOLEDO, Zurich, Switzerland). The original weight of the samples was weighed before the neutral salt spray test. After the neutral salt spray test, the sample was immersed in a de-rusting solution (the de-rusting solution used was composed of 37% AR hydrochloric acid and distilled water in a ratio of 1:1) for ultrasonic cleaning for 1 min to remove corrosion products, followed by cleaning with deionized water and alcohol. Weighing the sample after de-rusting and drying, the final corrosion weight loss was calculated by the following formula:

(1) $$v=\frac{m_{1}-m_{2}}{S\cdot t}$$

*v* is the Diamond/Cu composite sample corrosion rate (g/(cm^2^·h));
*t* is the corrosion time (h);
*m*_1_ is the original weight (g);
*m*_2_ is the weight (g) of the sample after the neutral salt spray test and de-rusting;
*S* is the area (cm^2^) of the Cu matrix exposed to the corrosive medium.

In this paper, Image-Pro Plus 6.0 was used to calculate the exposed copper matrix area of the Diamond/Cu composites. The copper matrix areas of 60 vol% and 75 vol% Diamond/Cu composites accounted for 53.0% and 24.2% of the sample surface, respectively.

### 2.4. Characterization

The corrosion morphology of the composite was observed using a scanning electron microscope JSM-7610FPlus of Hitachi, Tokyo, Japan. The extended depth of the corrosion interval of Diamond/Cu composites was obtained using a micro-area scanning electrochemical workstation (Ametek, Berwin, PA, USA, Versascan). The scanning setting was X-Y area, the scanning range was 6 × 6 mm, each step was 100 μm, and the probe size used for the surface scanning was 100 μm. The thermal diffusivities of the Diamond/Cu composites at room temperature were measured by a LFA447 thermal conductivity tester of NETZSCH Company (Selb, Germany), and the sample size had a diameter of 12.6 mm and a thickness of 2.5 mm. The bending strength of the samples was tested using an electronic universal material testing machine (AG-250KNIS, Shimadzu, Kyoto, Japan) with a displacement rate of 0.5 mm/min. The composition and structure of the corrosion products of the samples were characterized by X-ray diffraction (XRD) and energy dispersive X-ray spectroscopy (EDS).

## 3. Results and Discussion

### 3.1. Surface Morphology and Microstructure

**Figure 1:**
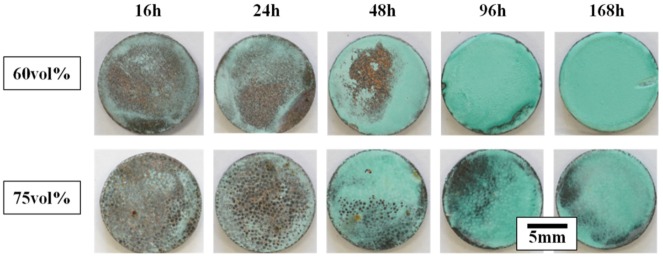
Photographs of the Diamond/Cu composites corroded surfaces after salt spray testing for different times.

Figure 1 shows the surface macro-morphology of Diamond/Cu composites after different times in a neutral salt spray environment. Visual inspection of the samples after the 168-h test revealed that the surface of the Diamond/Cu composites was completely corroded. The light green substance on the sample surface is the corrosion product. With the extension of corrosion time, the corrosion products on the surface of the Diamond/Cu composites increased continuously. The corrosion phenomenon mainly occurred on the copper matrix, and the diamond and interface products did not corrode [22]. When the neutral salt spray test lasted for more than 96 h, the corrosion products covered the entire surface of the Diamond/Cu composites. The corrosion products of 75 vol% Diamond/Cu composites were significantly less than those of 60 vol%. This is related to the exposed copper matrix area on the sample surface. The copper matrix area of 60 vol% and 75 vol% Diamond/Cu composites accounted for 53.0% and 24.2% of the sample surface calculated by Image-Pro Plus. Since only copper corrodes, the amount of corrosion products depends on the magnitude of the exposed copper matrix area.

**Figure 2:**
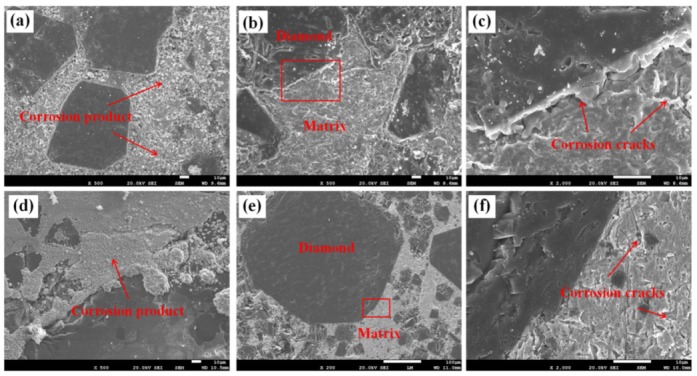
SEM image of 60 vol% Diamond/Cu composites containing (**a**) and removing (**b**) corrosion products; (**c**): partial enlargement of (**b**); SEM image of 75 vol% Diamond/Cu composites containing (**d**) and removing (**e**) corrosion products; (**f**) partial enlargement of (**e**).

Figure 2 shows the micro-morphology of the Diamond/Cu composites including and excluding corrosion products after the 48-h test. Corrosion products are distributed on the substrate in a granular form, as shown in Figure 2a,d. The black contrast is diamond and the gray contrast is copper matrix, as shown in Figure 2b,e. Diamond particles and interface product Cr_3_C_2_ (confirmed by previous research) were substantially free of corrosion [6]. However, discontinuous corrosion sites and cracks appeared on the copper matrix, indicating serious corrosion of the copper matrix, especially at the interface. This can be attributed to the differences in the physical and chemical properties of the materials and the heterogeneity of the Cu-Cr solid solution composition at the interface [8,16].

**Figure 3:**
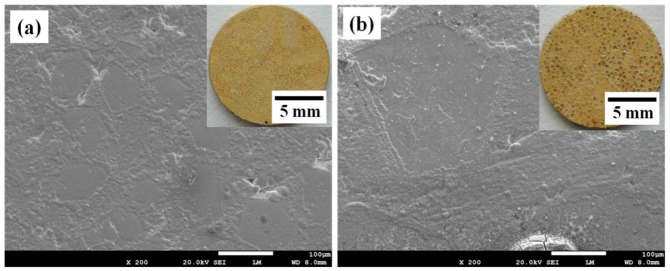
Optical images and SEM images of the gold-plated Diamond/Cu composites corroded surfaces after salt spray corrosion for 168 h ((**a**) 60 vol%; (**b**) 75 vol%).

Optical images and SEM images of the gold-plated Diamond/Cu composites’ corroded surfaces after salt spray corrosion for 168 h are shown in Figure 3. The gold-plated layer on the surface of the Diamond/Cu composites was uniform and compact. No corrosion products were observed on the sample surface. This indicates that gold plating on the surface can significantly improve the corrosion resistance of Diamond/Cu composites’ surfaces.

### 3.2. Corrosion Depth and Corrosion Rate

**Figure 4:**
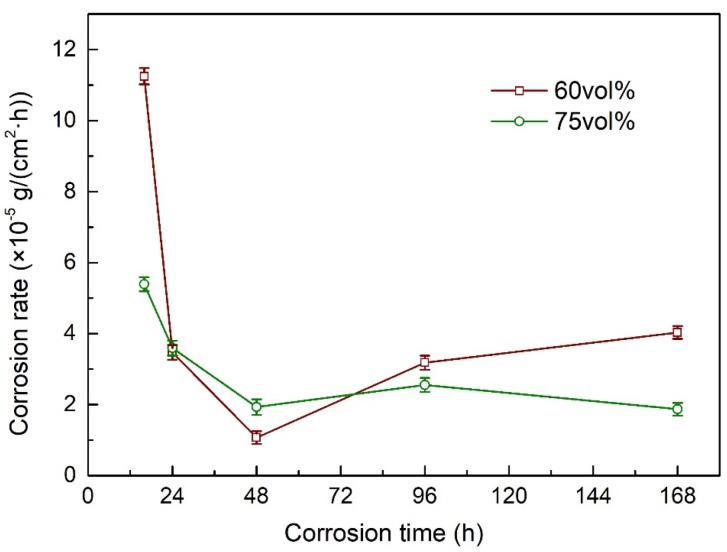
Corrosion rate of Diamond/Cu composites with different diamond content in neutral salt spray test.

Considering that diamond does not participate in corrosion, the area of Cu exposed was normalized. The corrosion weight loss curves of Diamond/Cu composites with different diamond content after a 5 wt% NaCl neutral salt spray test are shown in Figure 4. The corrosion rate of Diamond/Cu composites with different diamond contents decreased rapidly at first, and then stabilized after 48 h corrosion. This indicates that in the initial stage of corrosion, the generation of corrosion products inhibited the corrosion occurrence. At the initial stage of corrosion, the corrosion rate of 60 vol% Diamond/Cu composites was higher than that of 75 vol% Diamond/Cu composites. As the corrosion progressed, the corrosion rates of the two were similar. This indicates that the copper matrix corrosion rates of Diamond/Cu composites with different diamond contents are close.

**Figure 5:**
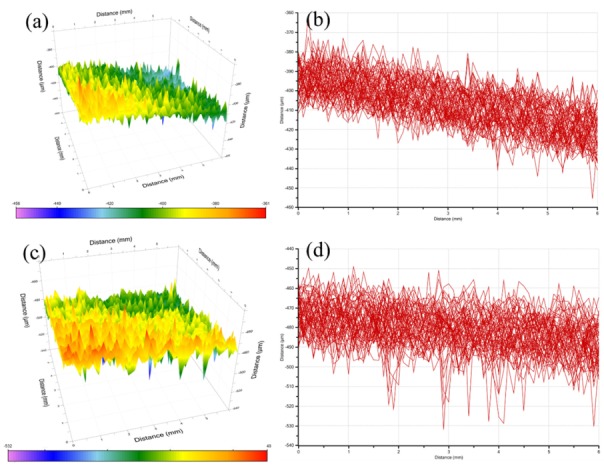
Corrosion depth of Diamond/Cu composites after salt spray corrosion for 168 h: (**a**) 3D topography of 60 vol% Diamond/Cu composites; (**b**) corrosion interval depth of 60 vol% Diamond/Cu composites; (**c**) 3D topography of 75 vol% Diamond/Cu composites; (**d**) corrosion interval depth of 75 vol% Diamond/Cu composites.

As the probe swept across the sample, the sample surface and corrosion cracks produced different signals. Corrosion depth can be calculated by the height difference of multiple scans in Figure 5b,d. The average of multiple scanning depths was the average corrosion depth. The extended depth of the corrosion pits of Diamond/Cu composites obtained by the micro-scanning electrochemical workstation is shown in Figure 5. The mean corrosion depths of composites with different diamond contents were similar. The average corrosion depths of 60 vol% and 75 vol% Diamond/Cu composites were 30 and 35 μm, respectively. As can be seen from Figure 5c,d, there are more sharp peaks in the OSP (Non-Contact Surface Profiling) scan results. It shows that 75 vol% Diamond/Cu composites have more and deeper corrosion sites, indicating that 75 vol% Diamond/Cu composites have more severe local corrosion.

### 3.3. Performance after Tests

**Figure 6:**
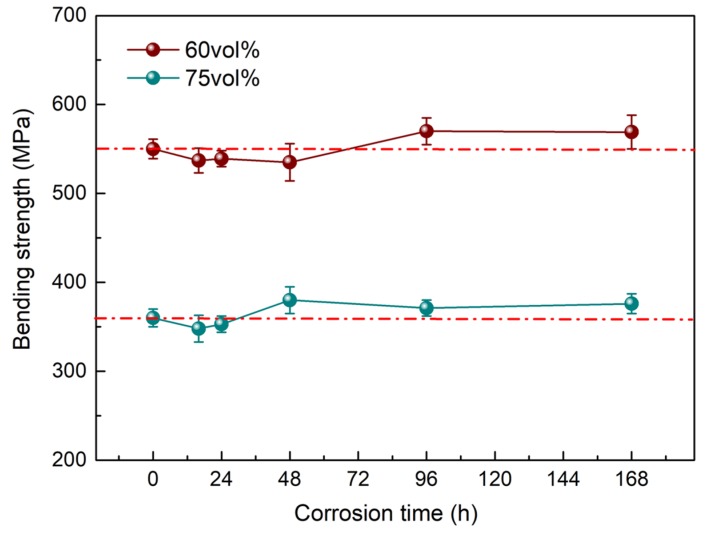
Bending strength of Diamond/Cu composites during neutral salt spray test.

The bending strength of Diamond/Cu composites after the neutral salt spray test is shown in Figure 6. With the prolongation of corrosion exposure time, the strength of the Diamond/Cu composites fluctuated around the initial value (red dashed/dotted line) before the test, and the fluctuation range was less than 5%. There was no significant increase or decrease in the bending strength of Diamond/Cu composites as the corrosion progressed. Therefore, it can be considered that the change in bending strength was mainly caused by the difference of the sample itself. The neutral salt spray test had little effect on the bending strength of Diamond/Cu composites. This was consistent with the performance change of Diamond-Cu composites in a humid environment [17,23].

**Figure 7:**
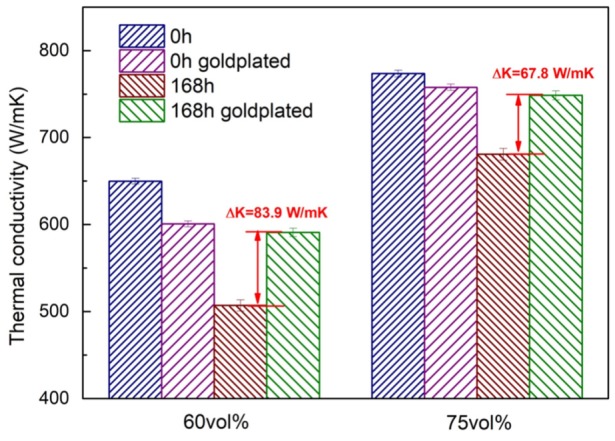
Thermal conductivity of Diamond/Cu composites before and after 168-h neutral salt spray test.

As shown in Figure 7, after the 168-h neutral salt spray test, the thermal conductivity of 60 vol% and 75 vol% Diamond/Cu composites without surface treatment decreases by 142.9 and 92.8 W/mK, and the decay rates reach 22% and 12%, respectively. The thermal conductivity of gold-plated Diamond/Cu composites decreased by 9.8 and 9.2 W/mK, respectively. The effect of neutral salt spray corrosion on the thermal conductivity of Diamond/Cu composites was mainly caused by surface roughness, such as corrosion cracks on the surface of the sample after corrosion. It did not affect the interface bonding state of Diamond/Cu composites. As a result, the thermal conductivity showed a slight decrease, down 1.6% and 1.2%, respectively. After the gold plating treatment, although the partial thermal conductivity of the Diamond/Cu composites was lowered, the surface state and performance after corrosion of the composite were greatly improved.

### 3.4. Corrosion Mechanism

Due to the different physical and chemical properties of diamond and metal substrates, there were many interfacial regions with different properties. In addition, the surface roughness of the sample after mechanical polishing was *Ra* = 1–2 μm measured by roughness meter: higher roughness resulted in smaller pits on the matrix surface. These places would provide preferential attack in the salt spray environment. Corrosion pits first appeared at the interface and the pits of matrix, and finally some corrosion crevices formed [24]. Therefore, the corrosion mechanism of Diamond/Cu composites was micro-galvanic corrosion.

**Figure 8:**
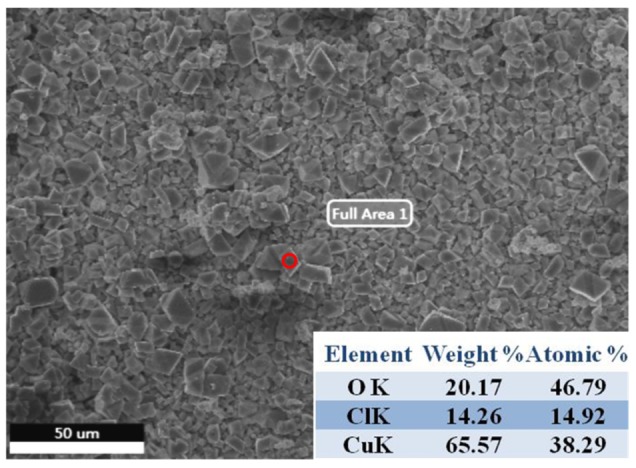
EDS analysis of the corrosion products.

**Figure 9:**
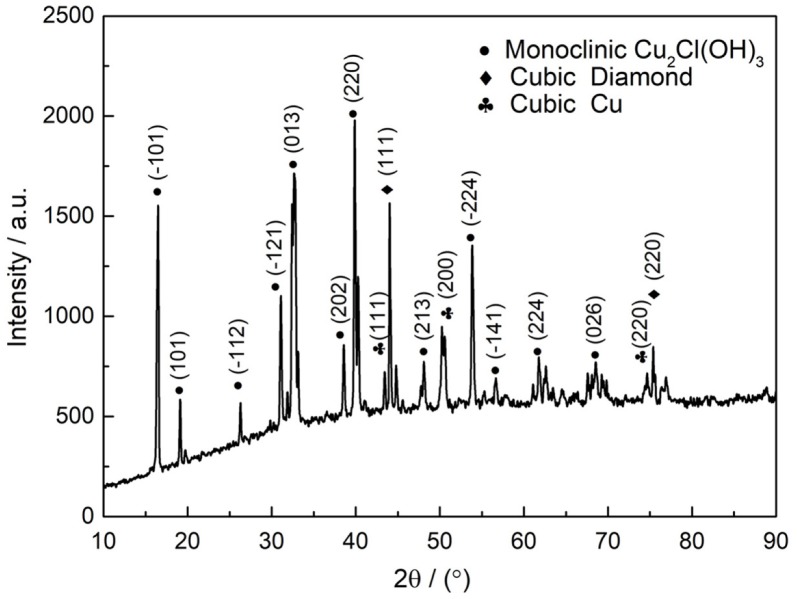
XRD pattern of Diamond/Cu composites corrosion products.

The EDS analysis of the corrosion products of Diamond/Cu composite samples after 168-h corrosion showed that the corrosion products contained O, Cl, and Cu elements (Figure 8). The corrosion product is confirmed to be Cu_2_Cl(OH)_3_ by XRD as shown in Figure 9. The electrochemical reaction that occurred was analyzed based on the corrosion product.

Anode reaction: Cu → Cu^2+^ + 2e^−^

Cathodic reaction: 1/2O_2_ + H_2_O + 2e^−^ → 2OH^−^

Salt spray is a dispersion system consisting of many tiny droplets of sodium chloride. When the Diamond/Cu composite was in this environment, it was easy to form a thin water film containing a large amount of sodium chloride on the surface of the sample, and the sample was subjected to electrochemical etching. Matrix copper entered the solution in the form of hydrated ions, leaving electrons in the metal, which flowed from the anode to the cathode. O_2_ reached the cathode surface by diffusion or convection to absorb the remaining electrons in the metal to form OH^−^. Cu^2+^ formed corrosion product Cu_2_Cl(OH)_3_ with Cl^−^ and OH^−^ in the solution [16,25].

The electrode reaction and corrosion process can be inferred by determining the composition of corrosion products. The salt spray formed an electrolyte film on the sample surface, which provided the necessary conditions for electrochemical corrosion. O_2_ in solution played an important role in the corrosion process. At the initial stage of corrosion, the sample surface was completely exposed. O_2_ easily reached the metal surface through diffusion, resulting in a faster corrosion rate. With the increase of corrosion products, the diffusion of O_2_, Cl^−^ was limited, and the corrosion rate decreased. Eventually diffusion reached a dynamic equilibrium, and the corrosion rate remained essentially unchanged.

## 4. Conclusions

Diamond/Cu composites were prepared by pressure infiltration. This paper investigated the thermal conductivity, mechanical properties, and corrosion behavior of the Diamond/Cu composites in a neutral salt spray environment. Based on the experimental results, the following conclusions were drawn:

1. A salt spray environment will seriously damage the surface morphology of Diamond/Cu composites. The corrosion product can cover the entire surface after 48-h tests. Localized corrosion is induced along the diamond-matrix interface. As the corrosion time prolongs, microcracks are created at the interface, and then the corrosion begins and propagates throughout the surface of the composite.

2. After the 168-h neutral salt spray test, the thermal conductivity of 60 vol% and 75 vol% Diamond/Cu composites without surface treatment decreased by 142.9 and 92.8 W/mK, respectively. The thermal conductivity of gold-plated Diamond/Cu composites decreased by 9.8 and 9.2 W/mK, respectively. There was no significant increase or decrease in the bending strength of Diamond/Cu composites as the corrosion progressed. The strength of Diamond/Cu composites fluctuated less than 5% compared to that of the initial value.

3. Surface metallization is an effective measure to improve the corrosion resistance of Diamond/Cu composites in a neutral salt spray environment, and can effectively improve the surface state and thermal conductivity of the composite.

4. The corrosion mechanism of Diamond/Cu composites is micro-galvanic corrosion. The corrosion product formed is Cu_2_Cl(OH)_3_.
